# P76 International passenger mobility and carbapenem-resistant *Klebsiella pneumoniae* in England, 2021–24: a time-series case study analysis

**DOI:** 10.1093/jacamr/dlaf230.083

**Published:** 2025-12-04

**Authors:** Sin Hang Phoenix Hui

**Affiliations:** Division of Medical Education, School of Medical Sciences, Faculty of Biology, Medicine and Health, University of Manchester, Manchester, UK

## Abstract

**Background:**

Antimicrobial resistance (AMR) poses a major threat to public health globally, with carbapenem-resistant *Klebsiella pneumoniae* (CRKP) of particular concern in the UK. Carbapenems are also classified as ‘Reserve’ antibiotics under the WHO AWaRe framework, reflecting their critical role as last-line agents where few alternatives exist. Rising resistance in these agents is therefore a priority target for stewardship and surveillance. England is a hub of international mobility, with passenger flows concentrated in London and major regional airports. While case reports have linked resistant infections to travel, quantitative evidence on how fluctuations in passenger volumes influence resistance at the regional level remains limited. Addressing this gap is essential to support UKHSA surveillance priorities and inform infection-prevention strategies in high-mobility settings.

**Objectives:**

The objective of this study was to quantify the relationship between international passenger mobility and AMR, using CRKP as a case study across English regions from 2021 to 2024. Specifically, we aimed to determine whether increases in passenger flows were followed by measurable changes in resistance levels, and to assess the temporal lag of this association.

**Methods:**

We constructed a panel dataset of nine English regions (2021–2024) combining monthly Civil Aviation Authority passenger volumes with UK Health Security Agency (UKHSA) surveillance of CRKP. The primary outcome was the percentage of resistant isolates per region-month. Fixed-effects linear regression models were estimated, with resistance as the dependent variable and passenger volumes (millions) as the predictor. Specifications included pooled ordinary least squares, region and month fixed effects, and lag structures of 1–6 months. Standard errors were clustered at the regional level to account for autocorrelation.

**Results:**

Analysis of 432 monthly observations (nine English regions, 2021–2024) was performed, linking Civil Aviation Authority passenger flows with UKHSA surveillance of CRKP. In pooled ordinary least squares, each additional million passengers were associated with a 0.13 percentage-point increase in CRKP resistance (95% CI 0.09–0.17, *P<*0.001; R²=0.51). In two-way fixed-effects models controlling for regional heterogeneity and month-specific shocks, the association persisted (β=0.19, 95% CI 0.09–0.29, *P*=0.003). Lagged models indicated that the effect was strongest at one month (β=0.18, 95% CI 0.07–0.29, *P*=0.01) and weaker at three months (β=0.16, 95% CI −0.01–0.33, *P*=0.06), while no association was detected at six months. In distributed lag models including contemporaneous and lagged flows simultaneously, coefficients were imprecise, reflecting multicollinearity between successive traffic measures.

**Conclusions:**

Increases in passenger mobility were associated with higher CRKP resistance within 1–3 months, consistent with importation and early dissemination of resistant strains. These findings underline the importance of strengthening AMR surveillance systems in regions with high travel volumes and suggest that monitoring mobility data could provide an early signal for emerging resistance trends. More broadly, this approach provides a transferable framework for analysing other AMR pathogens and drug-resistance combinations.

